# Temporary carbon dioxide removals to offset methane emissions

**DOI:** 10.1038/s41558-025-02487-8

**Published:** 2025-12-05

**Authors:** Frank Venmans, Wilfried Rickels, Ben Groom

**Affiliations:** 1 Grantham Research Institute on Climate Change and the Environment, London School of Economics and Political Science, London, UK; 2 Kiel Institute for the World Economy, Kiel, Germany; 3 LEEP Institute, Department of Economics, University of Exeter Business School, Exeter, UK

**Keywords:** Environmental economics, Climate-change policy

## Abstract

Unlike CO_2_, methane emissions have a particularly large short-term effect on temperature. We argue that these largely temporary temperature effects of methane emissions are apt to be offset by temporary CO_2_ removal. Temporally matching offsetting temperature reductions to the temperature impulse of methane eliminates the sizable intertemporal welfare transfers that occur when methane is offset by equivalent permanent CO_2_ removals. Assessing equivalence based on avoided economic damages suggests that about 87 temporary CO_2_ removals over a period of 30 years are needed to offset 1 t of methane. Agreement on the appropriate quantity of temporary CO_2_ offsets is insensitive to controversial parameters such as the social discount rate, climate damages and future emission scenarios. Short-term monitoring periods of 20–30 years are likely to be more credibly enforceable for various nature-based CO_2_ removal projects than long-term monitoring requirements.

## Abstract (web summary)

Methane emissions have a large short-term impact on temperature, which can be potentially offset by nature-based solutions that provide temporary carbon storage. This research demonstrates such matching could minimize intertemporal welfare trade-offs and avoid various risks for permanent removal.

## Main

Anthropogenic methane (CH_4_) emissions are the second largest cause of climate change after carbon dioxide (CO_2_) emissions, contributing 0.5 °C (estimated range, 0.3–0.8 °C) to global warming between the preindustrial era and 2010–2019^1^. Unlike CO_2_, CH_4_ emissions have a particularly large short-term effect on temperature^2^. Various initiatives have been taken to reduce CH_4_ emissions, most notably the global methane pledge which aims to reduce CH_4_ emissions by at least 30% below 2020 levels by 2030, in particular targeting low abatement cost options in the energy sector^3,4^. However, about 40% of global CH_4_ emissions come from the agriculture, forestry and other land uses (AFOLU) sector^5^. Even in the most ambitious scenarios of the Sixth Assessment Report of the IPCC^6^, the minimum annual amount of CH_4_ emissions in the AFOLU sector is still about 33 MtCH_4_ by 2050^7^. At the same time, the AFOLU sector plays a crucial role in mitigating climate change by removing atmospheric CO_2_ to offset residual CO_2_ and other greenhouse gas (GHG) emissions. The AFOLU sector achieves this primarily through nature-based solutions (NBS), such as afforestation. These solutions often provide only temporary carbon storage, unlike permanent CO_2_ removals achieved by methods involving geological carbon storage such as direct air carbon capture and storage. While offsetting can result in net-zero GHG emissions in simulated emission scenarios based on a 100-year global warming potential (GWP), it fails on two fronts: near-term climate benefits of CH_4_ emission reductions are not achieved^8–15^; and the integration of offsetting with temporary CO_2_ removal (for example, afforestation) into emissions trading systems is not addressed.

Various advances have been proposed to improve the representation of short-lived climate forces, and of CH_4_ emissions in particular, in climate policies and carbon budget calculations^12–14^. In this study, we focus on offsetting residual CH_4_ emissions. We argue that offsetting the short-term warming effect of CH_4_ emissions with equivalent temporary CO_2_ removals has several practical advantages. First, temporary and temporally coincident CO_2_ removals better mitigate the large short-run temperature effect of CH_4_ emissions and smooth out the damages of climate change across generations. Second, short-term monitoring periods for CO_2_ removal are more credibly enforced (as part of the crediting process and the contractual documentation) compared with long-term monitoring periods, and more easily renegotiated in the event of under- or overperformance^16,17^. Third, even if NBS have a long-term effect, they can still be administered by short-term monitoring periods. If the project has still removed carbon compared with a well-defined counterfactual at the end of the initial monitoring period (that is, it is still additional), the same project can compensate other CH_4_ emissions. Indeed, 20- to 30-year contractually agreed monitoring periods are used in the economy at large (for example, mortgages), are seen in policy (for example, biodiversity offsets in the United Kingdom^18^), and have the same duration as the main temperature effect of CH_4_ emissions. Finally, because both warming by CH_4_ emissions and cooling by temporary CO_2_ removal take place in the short run, the calculation of how much CO_2_ removal is equivalent to 1 t of CH_4_ emissions is insensitive to key determinants of intertemporal trade-offs of welfare: the social discount rate, economic damage parameters and the expected representative concentration pathway (RCP) scenario.

In terms of the value of damages avoided in the long run, we show that 1 t of CH_4_ emitted can be offset by between 78 and 117 temporary CO_2_ removals with a duration of 30 years across all scenarios presented, with 87 t CO_2_ in our central RCP 2.6 case. This narrow range illustrates the modest effect of assumptions about the discount rate, future warming and the failure risk within the 30 storage years.

## Matching schedules of CH_4_ emissions to temporary CO_2_ removals

Despite recent advances in comparing the climate change impact of CH_4_ emission rate changes to CO_2_ emissions pulses^12–14,19^, the most commonly applied metric to measure the impact of a GHG is still the global warming potential (GWP_*X*_), defined as the extra energy that is absorbed by the Earth as a consequence of 1 t of emission over a given number of years (*X*). Over 20 (100) years, the GWP of 1 t of CH_4_ is approximately 82.5 (29.8) times larger than 1 t of CO_2_. Values for non-fossil CH_4_ emissions are slightly lower (79.7 and 27.0 respectively) because the carbon atom of CH_4_ originates from atmospheric CO_2_ (IPCC WGI Table 7.15)^20^. The difference in GWP_*X*_ between CH_4_ and CO_2_ reflects their different energy forcing and how this forcing gradually dissipates over time. CH_4_ oxidizes to CO_2_ within decades, while CO_2_ is absorbed by oceans over centuries. As a result, when establishing GWP_*X*_ equivalence of CH_4_ and CO_2_ over *X* years (usually 100), the effect of CO_2_ beyond 100 years is ignored, making it hard to assess the dynamic trade-offs between both gases^8–13,15^.

The permanent removal of 1 t of CO_2_ has the same GWP as the emission of 1 t of CO_2_, but of opposite sign. A temporary removal will have a more modest GWP. Table 1 (last row) reports the number of temporary CO_2_ removals (each removing 1 t) with an equivalent GWP of 1 t of CH_4_ emitted. For example, 80 (25) CO_2_ removals over 30 (100) years offsets the GWP of 1 t of CH_4_. The equivalence ratios are slightly different from the IPCC ratios because we include the residual forcing effect after the CO_2_ removal and we assume rising concentrations in the future according to RCP 2.6, whereas the standard protocol of the IPCC assumes constant 2014–2019 GHG concentrations.

**Table 1:** Equivalence table for temporary and risky CO_2_ offsets to CH_4_ emissions

RCP	Discount rate	Failure risk	Duration of equivalent offset (years)						
	*r*	*ϕ*	20	25	30	35	40	100	500
2.6	2.5%	0.0%	132	105	87	75	66	30	17
2.6	2.5%	0.5%	140	113	95	83	74	38	27
2.6	2.5%	1.0%	148	121	103	91	81	46	38
2.6	3.0%	0.0%	119	96	81	71	63	32	23
2.6	3.0%	0.5%	126	103	88	77	69	39	32
2.6	3.0%	1.0%	133	110	95	84	76	48	42
2.6	3.5%	0.0%	112	92	78	69	62	35	29
2.6	3.5%	0.5%	118	98	84	75	68	42	38
2.6	3.5%	1.0%	125	104	91	82	75	50	47
4.5	2.5%	0.0%	136	107	88	75	65	26	10
4.5	2.5%	0.5%	151	120	100	86	76	34	19
4.5	2.5%	1.0%	162	131	110	96	85	44	31
4.5	3.0%	0.0%	124	99	82	71	62	28	16
4.5	3.0%	0.5%	133	107	91	79	70	36	26
4.5	3.0%	1.0%	141	115	98	86	78	44	36
4.5	3.5%	0.0%	116	94	79	69	61	31	22
4.5	3.5%	0.5%	123	101	86	76	68	39	32
4.5	3.5%	1.0%	130	108	93	83	75	47	42
6.0	2.5%	0.0%	142	111	91	77	66	25	9
6.0	2.5%	0.5%	161	128	106	91	79	34	18
6.0	2.5%	1.0%	174	140	117	102	90	44	30
6.0	3.0%	0.0%	129	103	85	73	64	27	14
6.0	3.0%	0.5%	140	113	95	82	72	35	24
6.0	3.0%	1.0%	149	121	103	90	81	44	35
6.0	3.5%	0.0%	121	97	82	71	62	30	21
6.0	3.5%	0.5%	128	104	89	78	70	38	30
6.0	3.5%	1.0%	135	112	96	85	77	46	41
GWP	0.0%	0.0%	120	96	80	69	60	25	5

**Fig. 1:**
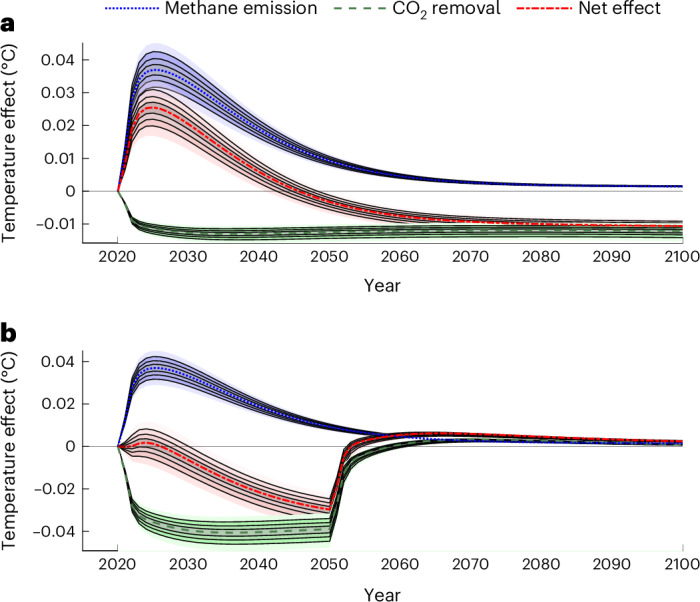
The temperature effect of a CH_4_ emission, offset by 25 100-year CO_2_ removals and by 80 30-year CO_2_ removals. The effects on temperature are estimated using the FAIR 2.0.0 model^50^. **a** shows the effect on temperature of a CH_4_ emission offset by 25 100-year CO_2_ removals. **b** shows the same for 80 30-year CO_2_ removals. In each panel the blue line is the impulse response function for 1 Mt of CH_4_ emission. The green line reflects the effect of the temporary CO_2_ removals. The red line charts the temperature change when the CH_4_ emission is offset by 25 100-year CO_2_ removals or 80 30-year CO_2_ removals (GWP equivalence ratio for RCP 2.6, including forcing effects after the end of the offset). Deciles represent physical uncertainty regarding gas forcing, absorption and decay dynamics (Supplementary Section 1 provides details of the temperature impulse response functions, while Supplementary Section 3 provides details of the physical uncertainty).

Welfare equivalence is calculated by first choosing the duration of 1-t offsets, then calculating how many of such offsets have the same welfare effect in terms of damages avoided. This is calculated by taking the ratio SVM/SVO for each duration of offset. Assuming a quadratic damage function, we show that the equivalence ratios do not depend on the slope of the damage function (Methods). The assumed growth rate of GDP is 2%, with GDP_2023_ = US$106 trillion (World Bank). Underlined values indicate the duration of the offset with the smallest welfare transfer, measured by the absolute value of the area between the red line and the *x* axis in Fig. 1b. See Supplementary Table 1 for more details. The last line reports the equivalence in terms of the global warming potential (GWP equivalence), including the residual forcing effect after the end of the carbon removal project under RCP 2.6, rather than truncating at 100 years.

The temperature change resulting from an offsetting strategy for CH_4_ emissions with 100-year CO_2_ removals changes over time (Fig. 1a). The temperature impact of an emission of 1 t of CH_4_ rises quickly and then dissipates almost as quickly (blue line). This is offset by the temperature path arising from a project removing 25 t of CO_2_ for 100 years (green lines). The net effect on temperature when CH_4_ emissions and CO_2_ removal happen simultaneously is essentially a vertical summation of these two effects (red line). Because CO_2_ and CH_4_ have a different impact over time, warming is not perfectly offset, despite GWP equivalence. Initially there is a large increase in temperature, offset by a small reduction in temperature in the long run. This offsetting strategy, and its path of temperature changes, involves a welfare transfer from present to future populations because damages increase in the short term and decrease in the long term.

An alternative strategy is to offset 1 t of CH_4_ emissions with a project that removes 80 t of CO_2_ for 30 years (Fig. 1b). By offsetting CH_4_ emissions with temporary CO_2_ removals, the mismatch in temporal horizons between CO_2_ and CH_4_ forcing is much smaller. This reduces the peak temperature effect and consequently the extent of the intertemporal welfare transfers. The net temperature effect (red line) shows a very small temperature increase in the very short run (5 years), followed by a temperature reduction until 2050, followed by a small and declining increase in temperature after release. The temporary strategy smooths out, although not perfectly, fluctuations in temperature compared with the long-term strategy (Fig. 1a), and therefore reduces intertemporal welfare effects while maintaining equivalence in terms of GWP in both scenarios (Fig. 1a,b). The number of equivalent temporary projects varies with the length of the storage period, with more 1-t projects required to achieve equivalence for shorter storage periods. Storage periods of 20 years^12^ or 40 years could also be chosen for removal projects, depending on physical properties, monitoring or financing requirements (Supplementary Figs. 1 and 2).

## Welfare equivalence between temporary CO_2_ removals and CH_4_ emissions

GWP-equivalent offset strategies for CH_4_ are not unique, leading to different temperature profiles and intertemporal transfers of welfare. An explicit treatment of the welfare effects of CO_2_ and CH_4_ emissions is therefore useful to indicate the types of trade-off that society finds acceptable and equivalent in welfare terms, that is, welfare equivalent. The welfare effects of a 1-t pulse of CO_2_ and CH_4_ emissions are measured by the present value of the damages they cause, respectively known as the social cost of carbon (SCC) and the social cost of methane (SCM). Different time profiles of the temperature effects of each gas lead to different marginal damages and social costs, so the SCC (for example, Dietz et al.^21^) and the SCM (for example, Azar et al.^22^) differ considerably. The ratio of these quantities can be used to establish the welfare equivalence of CO_2_ and CH_4_, without the need to limit the time horizon of analysis, as is necessary with GWP. With some sensitivity to modelling parameters as a result (for example, discount rate, damage parameters)^21^, SCM/SCC indicates how much more damaging a pulse of CH_4_ is to welfare compared with a pulse of CO_2_. Similar welfare-related measures are available for temporary CO_2_ removals, such as the social value of an offset (SVO)^17^, which is a well-defined fraction of the SCC. Formal definitions of the SCM, SCC and SVO are given in the Methods. Combinations of these measures allow welfare equivalence to be estimated for permanent and temporary offsetting strategies associated with CO_2_ and CH_4_ like those shown in Fig. 1.

## Estimates of welfare effects for CH_4_ and CO_2_

Early estimates, applying the PAGE integrated assessment model, found a SCM between $40 and $414 per tCH_4_ (in 2020 prices)^23,24^. Subsequent studies found considerably higher SCMs estimates of $710 per tCH_4_ (ref. ^25^), $1,350 per tCH_4_ (ref. ^26^) and $1,950 per tCH_4_ (ref. ^27^) (2020 prices in US$, 2.5–3% discount rate). In 2023, the US Environmental Protection Agency^28^ estimated results for three damage specifications: the Data Driven Climate Impact Model (DSCIM)^29–31^, the Greenhouse Gas Impact Value Estimator (GIVE) model^32^ and a recent meta-analysis^33^, coupled with three near-term discount rates, 1.5%, 2.0% and 2.5%. The mean SCM ranges between $470 per tCH_4_ for the estimate obtained with DSCIM damages and 2.5% discount rate and $2,900 per tCH_4_ using the damages from the the meta-analysis and a 1.5% discount rate. Azar et al.^22^ find a considerably higher SCM of $8,075 per tCH_4_ (with a near-term discount rate of 2.5%) in their business-as-usual emissions scenario (which is similar to RCP8.5). However, it should be noted that the corresponding SCC estimate is $1,196 per tCO_2_ which is at the upper range of estimates found in a recent recent multimodel SCC synthesis that reports median and mean estimates of $62.61 per tCO_2_ and $250.87 per tCO_2_, respectively^34^ ($194.61 per tCO_2_ when excluding the lower and top 0.1% of the estimates from the study).

## Welfare equivalence of CH_4_ and CO_2_

Turning to the equivalence of CO_2_ and CH_4_, the estimate of Azar et al. results in a SCM/SCC ratio of about 7, which increases to 21 for their optimal policy scenario (in which the SCM and SCC drop to $3,997 per tCH_4_ and $192 per tCO_2_, respectively). The SCM/SCC ratio varies considerably with the convexity of the damage function with respect to temperature, the policy scenario (that is, BAU versus optimal policies) and the discount rate. For example, across the nine scenarios analysed by the Environmental Protection Agency, a ratio between 4 and 14 is reported, compared with early estimates that range between 21 (ref. ^23^) and 47 (ref. ^35^).

The sensitivity of SCM/SCC is problematic for implementation because it can lead to disagreement on operational values. Yet, offsetting CH_4_ with temporary CO_2_ removal reduces the sensitivity of welfare equivalence because shorter time horizons are considered. Here, the SVO is the relevant metric for establishing welfare equivalence, not the SCC^17^. The SVO measures the value of temporary CO_2_ removal as a well-defined fraction of the SCC, correcting for storage duration and storage failure risk. The relevant measure of equivalence for temporary CO_2_ and CH_4_ is SCM/SVO, indicating how many 1-t temporary CO_2_ removals are welfare equivalent to a 1-t impulse of CH_4_.

Table 1 shows the equivalence analysis using SCM/SVO to measure welfare equivalence, using equation (3) (Methods) to calculate the SVO. The results are shown for different time horizons for CO_2_ removals, ranging from 20 to 500 years. The equivalence is insensitive to horizons beyond 500 years, so a temporary removal of 500 years duration can be thought of as a permanent removal. Columns 1–3 show the RCP scenario, the discount rate (*r*) and the annual failure risk (hazard rate, *ϕ*) of a project, respectively. Columns 4–10 show the number of temporary CO_2_ removals that would be welfare equivalent to a 1-t CH_4_ emission in terms of damages offset by strategies of different durations. The equivalence ratios for permanent removals in column 11 for zero failure risk correspond to the ratio SCM/SCC. Assuming a quadratic damage function, we show that the equivalence ratios are independent of the slope of the damage function (Methods).

In row 1 we see that in RCP 2.6 the equivalent permanent CO_2_ removal is 17 t. This is different from the GWP_100_ ratio of 30 because the SVO approach takes into account the warming effect of CO_2_ after 100 years, thermal inertia and a non-linear damage function. This value more than doubles to 38 t when yearly failure risk increases from 0% to 1% (row 3): risk compounds over time and requires more removal projects to maintain welfare equivalence. Being welfare/damages related, welfare equivalence is sensitive to the discount rate, requiring more removal projects if the long-term cooling of CO_2_ removal is discounted at a higher rate. Equivalence in Table 1 also increases as one moves from permanent removals to shorter-run temporary removals, ranging from 17 t for permanent removal to 132 t for a 20-year project.

Although all offsetting strategies in Table 1 are welfare equivalent, each strategy entails different distributions of well-being over time. Each has different patterns of compensation between medium-term welfare gains from lower temperatures and losses from slightly higher temperatures in the long run (for example, compare the paths of temperature changes for the 30-year strategy in Fig. 1b and the 20- and 40-year strategies in Supplementary Fig. 1). For each row in Table 1, the underlined value is the duration with the smallest intergenerational welfare transfers as measured by adding up the absolute value of welfare gains and losses over time. More details on this calculation are given in the Methods. Supplementary Table 1 describes the optimal duration of removals and the minimum welfare transfer. For most future emission scenarios, discount rates and failure risks, the optimal removal duration is 30 years.

Comparing permanent and temporary removals within the 30-year column illustrates the insensitivity of equivalence to both the discount rate, yearly failure risk and the choice of the emissions scenario. For temporary 30-year projects, the equivalence ranges from 78 (RCP 2.6, discount rate 3.5%, failure risk 0%) to 117 (6.0, 2.5%, 1%), which is a relatively modest increase of 50% (and merely 16% for riskless projects). By contrast, the equivalence of an offset programme using permanent removals varies from 9 to 47, a 420% increase depending on the discount rate, risk level and emission scenario selected. Supplementary Section 3 reports results for the physical uncertainty (forcing and decay of both CH_4_ and CO_2_), showing that shorter removal projects have slightly lower physical uncertainty.

A comparison of the SCM/SCC and the SCM/SVO welfare equivalence with GWP equivalence is shown in the final row of Table 1. The equivalence of 5 for 500 years storage duration (column 10) reflects the fact that a permanent CO_2_ removal causes a permanent reduction in GWP. For infinite horizons the equivalence converges to 1 for fossil CH_4_ and to 0 for non-fossil CH_4_. The long-run effect of a non-fossil CH_4_ emission is 0; a pulse of CO_2_ emissions leads to a new long-term chemical equilibrium with more CO_2_ in the ocean and atmosphere, resulting in permanent residual forcing. A permanent CO_2_ removal does the opposite. In practice, then, measured using GWP rather than damages, any microscopic yet permanent removal of CO_2_ is equivalent in the long run in GWP terms to an emission of CH_4_. Over an infinite horizon, GWP equivalence is therefore approximately 0. This singularity result is a natural consequence of using a zero discount rate for future CO_2_ removals, hence the need for arbitrary definitions of permanence (for example, 100 years). Welfare equivalence is a theoretically grounded alternative.

## Discussion

Robust and practical carbon accounting schemes are crucial for the development of international emissions and CO_2_ removal trading. Such schemes will become increasingly important for future net-zero GHG policies and in light of the latest text on offsets in Article 6 agreed at the 2024 climate COP meeting^36^. Failing to properly account for carbon storage over time is considered a major obstacle for the implementation of NBS to enhance atmospheric CO_2_ removal^37–39^. Nowhere is this more pertinent than in relation to measures interfering with the terrestrial biological carbon pool, which currently accounts for the overwhelming majority of countries’ active CO_2_ removal activities^40^.

Various accounting approaches aim to deal with the potential non-permanence of carbon storage. Additional measures such as buffer accounts attempt to ensure that issued credits have a permanent carbon storage collateral (for example, the Reversal Risk Buffer Pool Account in the Paris Agreement Crediting Mechanism^41^). These approaches and measures focus on the integration of CO_2_ removals into carbon markets where long-term storage is supposed to offset the long-term impacts of CO_2_ emissions^42,43^. A useful addition to carbon accounting is to view temporary carbon storage as appropriate for countering short-term climate impacts. Using potentially non-permanent CO_2_ removal projects to compensate for short-term climate impacts resulting from CH_4_ emissions is a good example. Temporally matched, temporary offset strategies for CH_4_ can better neutralize associated temperature effects and minimize intergenerational transfers and trade-offs. Short-term strategies remove the influence of the social discount rate (which reduces long-run damage valuations) on welfare equivalence, thereby avoiding a major source of disagreement (for example, ref. ^44^) and facilitating the design of clear strategies.

Importantly, baseline welfare equivalence requires 87 30-year 1-t CO_2_ removals projects, compared with 17 permanent ones, increasing the compensation ratio by a factor of about 5. Yet, many nature-based CO_2_ removal projects, for example, in forest-based measures including reduced deforestation, are reported to provide offsets at costs well below $20 per tCO_2_ (refs. ^40,45^), or even at negative costs if co-benefits are monetized^46^. Realization of these low-cost removal projects is often hampered by long-run risks and problems monitoring and verifying long-run carbon storage^47^. Such concerns about NBS have limited the development of markets for these removals, despite recent voluntary market transactions showing that buyers are willing to pay a premium for the proven climate benefits of CO_2_ removal^48^. Repurposing temporary offsets to a more suitable purpose could improve their perceived quality, helping to capture these values. Thirty-year monitoring periods, which appear to minimize intertemporal trade-offs in the context of CH_4_, are also familiar from other contexts (for example, government bonds, mortgages). Monitoring could also occur at more frequent intervals throughout the 30-year duration as a governance requirement (as proposed elsewhere^16^). Furthermore, where NBS are additional for longer than 30 years, the same project could get certified again for a new period of 30 years, compensating another CH_4_ emission. Such approaches to contractual documentation and monitoring will increase credibility and reduce insurance costs in the market. This is useful, because some so-called ‘residual’ CH_4_ emissions will persist beyond 2100, especially in agriculture. Finally, recent estimates of the social cost of methane suggest that it is upwards of $7000 ton^−1^ (ref. ^49^). At this value, many temporary removals approaches would be economically viable even at an equivalence rate of 87 t of temporary CO_2_ removal.

Naturally, a net-zero society will also require permanent removals, such as geological storage or mineral weathering^42,43^. The general proposition here is that there are advantages in matching the remedy to the problem. For the temporary effects of CH_4_ emissions, temporary CO_2_ removals have advantages. For permanent CO_2_ emissions, permanent CO_2_ removals (for example, geological storage, or repeated temporary projects) are more appropriate. Separate permit markets for each could allow both to flourish where they are most appropriate.

## Methods

The SCC, SCM and SVO concepts are formally described, followed by the optimal offset duration calculation.

### SCC, SCM and SVO

The theoretical framework is an adaptation of Groom and Venmans^17^ that incorporates insights from Azar et al.^22^ on the characterization of the SCM.

Assume total damages of global warming *T* are quadratic and proportional to the size of the economy *Y*, that is $Y={Y}_{0}\exp (\frac{-\gamma }{2}{T}^{2})$, with *γ* the slope of the marginal damage function. As a result, the marginal damage MD of extra warming is $\mathrm{MD}(t)=\frac{{\rm{\partial }}Y}{{\rm{\partial }}T}=\gamma Y(t)T(t)$. Call Δ*T*_CH4_(*t*) the temperature impact response function of a pulse of 1 t of CH_4_ emissions. This temperature impact response function corresponds to the blue line in Fig. 1. The SCM is the discounted sum of all marginal damages of the extra warming Δ*T* resulting from the pulse,

1 $$\mathrm{SCM}=\mathop{\int }\limits^{\infty }_{0}\exp (-rt)\Delta {T}_{\mathrm{CH}4}(t)\mathrm{MD}(t){\rm{d}}t,$$

where *r* is the discount rate according to the Ramsey rule including pure time preference and a wealth effect to capture proportional changes in marginal utility in the future (higher consumption, lower marginal utility, higher discount rate). See ref. ^44^ for details.

Similarly, the SVO can be calculated using the temperature impulse response function of the temporary carbon removal, Δ*T*_CO2_(*t*), defined as a positive deviation from the baseline temperature. This temperature impulse response function corresponds qualitatively to the the green line in Fig. 1 (although the figure displays the response for 80 t). The SVO is the discounted sum of all marginal damages avoided by the temporary cooling from the offset,

2 $$\mathrm{SVO}=\mathop{\int }\limits^{\infty }_{0}\exp (-rt)\Delta {T}_{\mathrm{CO}2}(t)\mathrm{MD}(t){\rm{d}}t.$$

In the case of a risky removal project, we assume a constant failure rate *ϕ*, which leads to a likelihood of survival of $\exp (-\phi t)$ after *t* years. This boils down to increasing the discount rate with failure rate *ϕ* in the formula of the SVO.

Table 1 reports SCM/SVO for different parameter values and 1 t of each gas. Note that the damage function parameter *γ* does not affect this ratio, because it appears as a constant in both the numerator and the denominator. The other factors (GDP growth, background temperature *T* and discount factor) will have a limited effect to the extent that the impact response functions Δ*T*_CH4_ and Δ*T*_CO2_ mirror each other over time. Also, as marginal damages are proportional to GDP, the present value of the marginal damages is exp(−(*r* − *g*)*t*)*Y*_0_*γT*_*t*_, so the discount rate is reduced by the growth rate of GDP, leading to a very low ‘effective discount rate’ (0.5% in our baseline example). The derivation of optimal offset duration with respect to minimizing the net welfare transfer is detailed in Supplementary Section 2.

Note that the temperature impulse response function is close to a step function with 3 years of delay, due to thermal inertia. The step function with a delay of *ξ* years is in line with the common assumption that warming is proportional to cumulative CO_2_ emissions (*S*) between the preindustrial period and time *t*: *T*_*t*+*ξ*_ = *ζS*_*t*_, where *ζ* is the transient climate response to cumulative emissions (TCRE). The TCRE is remarkably stable over time and across emission scenarios^21,51,52^.

The approximation allows us to provide simple code where practitioners can set their tailored project duration and calculate the value of projects with gradually increasing (and decreasing) carbon removal. Call *q*(*t*) the quantity of carbon (in equivalents of CO_2_) stored by the project at each point in time. The SVO of the removal project is now

3 $$\mathrm{SVO}=\mathop{\int }\limits^{\infty }_{0}\exp (-r(t+\xi \,)-\phi t)q(t)\zeta \,\mathrm{MD}(t+\xi \,){\rm{d}}t.$$

Further details on the temperature and damage impulse response functions are given in Supplementary Section 1.

### Minimizing welfare transfers

The offset strategies that minimize welfare transfers are calculated as follows. Once the welfare equivalence is known, the absolute value of the area between the net damage function and the *x* axis is calculated (the area between red line in Supplementary Fig. 2 and the *x* axis). This is our measure of welfare transfers. The offset strategy with the duration of 1-t offsets that minimizes the welfare transfers (the sum of absolute deviations from the *x* axis) is selected as the optimal approach. These are the underlined strategies in Table 1. Supplementary Table 1 shows the welfare transfer as a percentage of the damages associated with the impulse of CH_4_.

### Reporting summary

Further information on research design is available in the Nature Portfolio Reporting Summary linked to this article.

## Online content

Any methods, additional references, Nature Portfolio reporting summaries, source data, extended data, supplementary information, acknowledgements, peer review information; details of author contributions and competing interests; and statements of data and code availability are available at 10.1038/s41558-025-02487-8.

## Supplementary information

Supplementary Information Supplementary Sections 1–3, References, Figs. 1–3 and Table 1.

Reporting Summary

## Data Availability

The data used to create the figures, tables and Excel spreadsheet are available via GitHub at https://github.com/FVenmans/OffsetMethane and Zenodo at 10.5281/zenodo.17228030 (ref. ^53^).

## Code availability

The code used to create the figures, tables and Excel spreadsheet are available via GitHub at https://github.com/FVenmans/OffsetMethane and Zenodo at 10.5281/zenodo.17228030 (ref. ^53^).
