# Supplementary material for: Temporary carbon dioxide removals to offset methane emissions
Source: Nat Clim Chang. 2025 Dec 5;16(1):37–42. doi: 10.1038/s41558-025-02487-8 (PMC12783049; doi:10.1038/s41558-025-02487-8)
Supplement: Supplementary file 1 — Supplementary Sections 1–3, References, Figs. 1–3 and Table 1. [file 41558_2025_2487_MOESM1_ESM.pdf]

nature climate change

Supplementary information https://doi.org/10.1038/s41558-025-02487-8

Temporary carbon dioxide removals to offset methane emissions

In the format provided by the authors and unedited

Contents

1 Temperature and damage impulse response functions for CH_4 and CO_2 2
2 Optimal duration of removal monitoring periods 5
3 Physical uncertainty of the equivalence ratio 7
References 7

1 Temperature and damage impulse response functions for CH_4 and CO_2

Our CO_2 and CH_4 impulse response functions are based on FAIR 2.0.0.[1] Natural CO_2 uptake is modeled with a '4 box model', based on Joos et al.[2] Each box has a different decay rate, acknowledging that different sinks absorb carbon at different speeds. It also acknowledges that emitting fossil CO_2 leads to a long term equilibrium where there is more carbon both in the oceans and in the atmosphere. Therefore, 22% of emissions are attributed to a box that is never absorbed. Moreover, the absorption speed depends on temperature and cumulative absorption since 1850. This represents the fact that warmer and more acidic oceans absorb CO_2 at a lower speed.

The decay of CH_4 is very different, because it is decomposed into water and CO_2 in the atmosphere. FAIR represents atmospheric CH_4 decay as a single reservoir system, resulting in exponential decay. This is more accurate for methane of biological origin than for methand of fossil origin.

Both CO_2 and CH_4 lead to climate forcing. Forcing is proportional to CH_4 concentration and proportional to the log of CO_2 concentration. A minor forcing effect is proportional to the square root of CO_2 concentration.

Forcing heats up the atmosphere and the upper layer of the ocean. This heat is then distributed to the lower oceans, represented by a '3 box model', similar to Geoffroy et al.[3] and DICE (which both have only 2 boxes). The distribution of heat results in thermal inertia and a fundamental difference between the temperature impulse response function and the forcing impulse response function. For example, for a pulse of CO_2, thermal inertia leads to a relatively constant warming effect over time, despite a declining forcing effect. This is because in later decades, although part of the CO_2 has already been absorbed, thermal inertia leads to a delayed warming effect from early forcing.

Figure S1 shows the temperature impulse response function for a pulse of 1 tonne of methane, compensated by the removal of 132 tonnes of CO_2 during 20 years (upper) and 66 tonnes of CO_2 during 40 years (lower). The red line shows the net temperature impact, as the impact of the CO_2 removal is relatively constant over time, whereas methane reaches its peak warming after a decade.

Figure S2 gives the present value of the net marginal damages from a CH_4 emission pulse and the marginal gains from the CO_2 removal project. Red line is the net welfare effect. To calculate the welfare effects, we assume a quadratic damage function of the form $D_t = GDP_t e^{\frac{\gamma}{2}T_t^2}$, with marginal damages $GDP_t \gamma T_t$. We calculate the total welfare effect as

$$\sum_{t=0}^{\infty} GDP_0 e^{-(r-g)t} \gamma T \Delta T, \tag{1}$$

where r is the discount rate, g is the growth rate of GDP and ΔT is the temperature impulse response function. In the case there is failure risk for the removal project, we assume a constant probability of failure ϕ. This results in a the likelihood of survival of $e^{-\phi t}$, which corresponds to adding ϕ to the discount rate for the removal project. The slope of the damage function γ has no effect on the equivalence ratio, because it appears both in the numerator and denominator.

The welfare effect of the tonne of methane is also known as the Social Cost of Methane (SCM), whereas the welfare effect of the removal project is the Social Value of the Offset (SVO) as in Groom & Venmans[4]. The equivalence ratio is then simply SCM/SVO. As a result, the cumulative positive net benefits of both projects (positive area above the x-axis) is equal to the cumulative negative net costs of both projects (negative area below the x-axis).

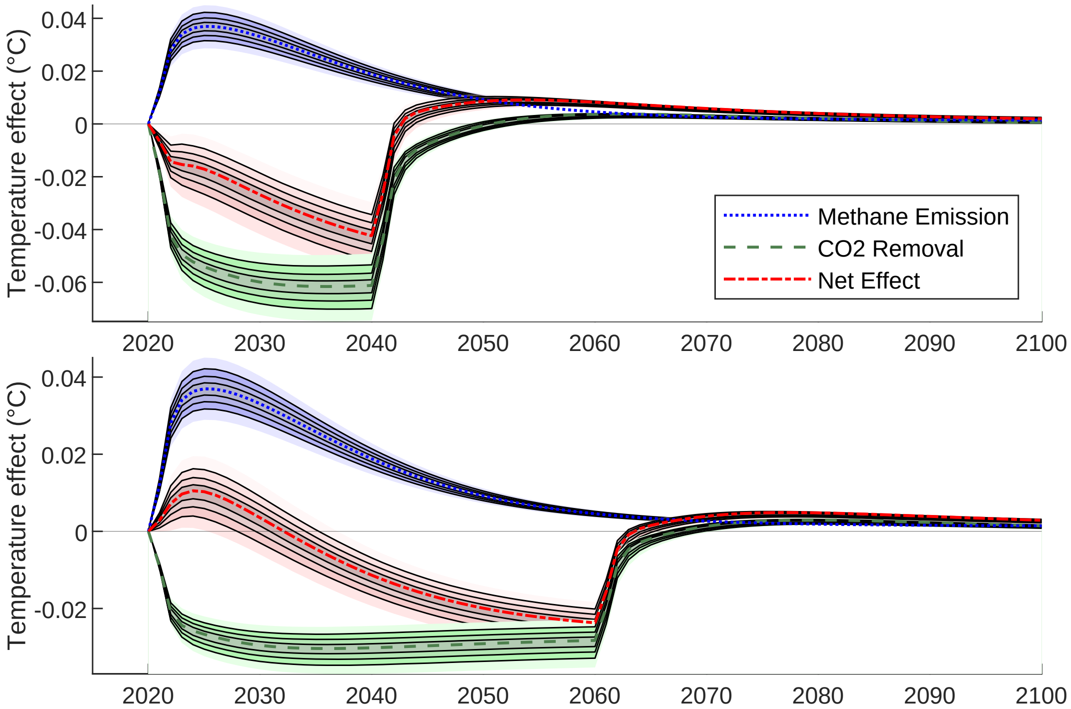

Figure S1. **Temperature impulse response function of CH_4 and CO_2 removal for 20 and 40 years.** The CH_4 impulse response function shows the difference between the temperatures of the SSP1 26 background scenario and the scenario with 1 Mt of methane added in 2020. The CO_2 impulse response function shows the difference between the temperatures of the SSP1 26 background scenario and a scenario instantaneously removing 132 $MtCO_2$ in 2020 and re-emitting it in 2040 (upper) and removing 66 tonnes in 2020 and re-emitting in 2060 (lower). Deciles represent uncertainty related to decay and forcing (see SM Section 3).

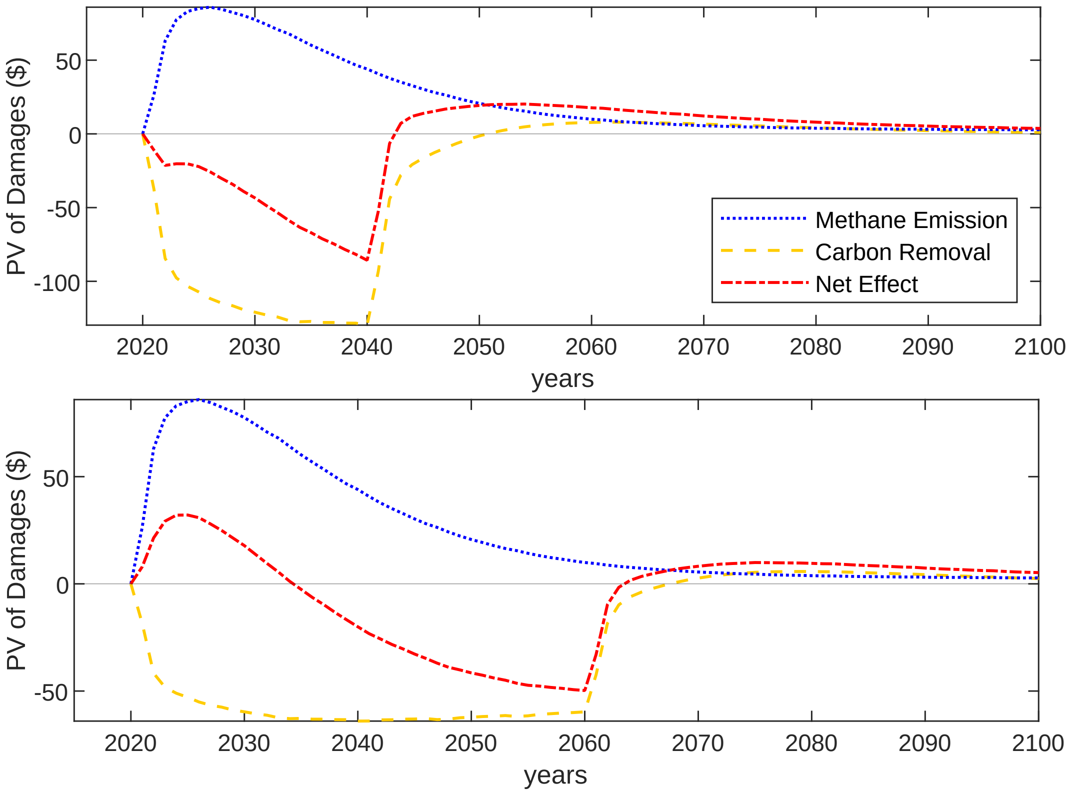

Figure S2. Welfare impact response functions of CH_4 and CO_2 removal for 20 and 40 years. Present value of marginal damages of an emitted tonne of CH_4 compensated by the removal of 132 tonnes of CO_2 over 20 years (upper) and 66 tonnes of CO_2 over 40 years (lower). Discount rate 2.5%, quadratic damages proportional to GDP, growth rate of GDP 2%, no failure risk, marginal damage slope $\gamma = 1.54\%$[5], GDP_{2023}=$106 Trillon (World Bank).

2 Optimal duration of removal monitoring periods

Table S1 shows for each carbon removal duration the net welfare transfers over time, adding up periods of positive welfare effects and the absolute value of negative welfare effects. For most settings, a carbon removal project of 30 years is the project with the lowest welfare transfers over time. For higher discount rates and higher risk, the welfare transfers are sometimes minimized at 25 year projects and for high temperatures and lower discount rates the optimal duration can increase to 35 years.

However, the table also shows that the welfare transfers are very similar for removal projects between 20 years and 40 years. This indicates that other considerations such as the incentive structure of project developers, monitoring, reporting, verification and the biological properties of the project should be important determinants of the optimal duration of projects. Note also that all offsets are designed to be welfare neutral. So longer offsetting projects are similar in nature to a welfare neutral investment by the current generation (improving the future), whereas shorter offset projects are similar to a welfare neutral debt.

RCP	Discount rate	Failure Risk	Duration of equivalent offset (years)						
		ϕ	20	25	30	35	40	100	400
2.6	2.5%	0.0%	78%	66%	64%	67%	70%	100%	108%
2.6	2.5%	0.5%	79%	66%	62%	64%	67%	91%	91%
2.6	2.5%	1.0%	78%	65%	59%	59%	62%	82%	77%
2.6	3.0%	0.0%	65%	55%	56%	60%	64%	94%	104%
2.6	3.0%	0.5%	64%	53%	53%	55%	59%	84%	88%
2.6	3.0%	1.0%	63%	50%	49%	51%	54%	74%	74%
2.6	3.5%	0.0%	55%	49%	51%	55%	60%	88%	97%
2.6	3.5%	0.5%	54%	46%	47%	51%	54%	78%	83%
2.6	3.5%	1.0%	54%	43%	43%	46%	49%	69%	70%
4.5	2.5%	0.0%	79%	66%	62%	63%	66%	96%	119%
4.5	2.5%	0.5%	87%	74%	66%	66%	68%	93%	97%
4.5	2.5%	1.0%	89%	76%	67%	65%	66%	85%	78%
4.5	3.0%	0.0%	70%	58%	57%	60%	63%	95%	115%
4.5	3.0%	0.5%	72%	58%	56%	57%	60%	87%	95%
4.5	3.0%	1.0%	72%	58%	53%	54%	56%	77%	78%
4.5	3.5%	0.0%	61%	52%	52%	56%	60%	91%	106%
4.5	3.5%	0.5%	61%	49%	49%	52%	55%	82%	89%
4.5	3.5%	1.0%	60%	47%	46%	48%	51%	72%	74%
6.0	2.5%	0.0%	80%	67%	62%	63%	65%	93%	116%
6.0	2.5%	0.5%	92%	80%	71%	69%	70%	91%	95%
6.0	2.5%	1.0%	96%	83%	74%	69%	68%	84%	76%
6.0	3.0%	0.0%	74%	61%	59%	60%	63%	93%	115%
6.0	3.0%	0.5%	77%	64%	58%	59%	61%	86%	95%
6.0	3.0%	1.0%	78%	64%	56%	56%	57%	77%	77%
6.0	3.5%	0.0%	65%	54%	54%	56%	60%	90%	107%
6.0	3.5%	0.5%	66%	53%	51%	53%	56%	81%	90%
6.0	3.5%	1.0%	66%	52%	48%	49%	51%	71%	74%

Table S1. Welfare transfers arising from offsetting strategy. The table shows welfare transfer effects as a proportion of the welfare cost of an emission of 1 tonne of CH_4. The welfare transfer effect is measured by the cumulative welfare effects over time, adding up the value of gains (lower damages) and the absolute value of losses (higher damages). This corresponds to the absolute value of the area between the red line in Figure S2 (the net welfare effect of methane emission minus offset) and the x-axis. Note that the number of 1-tonne CO_2 removal projects is chosen so that the total welfare effect of the emission and the offset (gains minus losses) is zero. That is, they are welfare-equivalent. The underlined values show the duration of the welfare-equivalent project that minimises the welfare transfer for a particular row. This corresponds to the underlined equivalence ratio shown in Table 1 in the main text.

3 Physical uncertainty of the equivalence ratio

Figure S3 shows the probability distribution of the equivalence ratio for a risk-less CO_2 removal project of 100 and 30 years on an RCP 2.6 scenario with a discount rate of 2.5% (row 1 in Table 1 in the main text). The uncertainty includes the uncertainty regarding the climate forcing of CH_4, the decay rate of CH_4, the climate forcing of CO_2, the absorption of CO_2, the sensitivity of this absorption to temperature, and the sensitivity of this absorption to cumulative absorption since 1850. The latter two effects matter because a warmer and a more acid ocean decreases the speed of absorption. The uncertainty is calibrated on the CIMP 6 modeling ensemble by the FAIR 2.0.0 team.[1] FAIR assumes these uncertainties to be independent, as they refer to very different physical processes. The uncertainty of methane decay comes from the 6th IPCC report, WGI (Table 6.2)[6], as it is not available in FAIR 2.0.0. We did not include uncertainties related to thermal inertia (equilibrium climate sensitivity and transient climate sensitivity) because these apply equally to methane and carbon forcing. Results are generated with 5000 Monte Carlo simulations.

Due to the non-linear effects of the uncertainty, the mean of the Monte Carlo equivalence ratio is approximately 3% higher and the median is 2% lower than the equivalence ratio of the estimate without physical uncertainty (using the average parameters). The mean equivalence of carbon for methane has a mean of 29.45 and 90.97 when calculated over 100 and 30 years respectively, as shown in Figure S3. This shows the importance of the horizon over which GWP equivalence is calculated. While the spread of the 30 year equivalence ratios appears wider for the 30 year horizon (in orange) than the 100 year horizon (blue), this is really an artefact of their different scales. The coefficient of variation (standard deviation divided by the mean: a normalised measure of spread that allows distributions of different scales to be compared) is lower for the shorter time horizon (0.33) compared to the longer time horizon (0.36). Consequently, there is more uncertainty in equivalence in the long run than in the short run offsets. The standard deviations for the 20, 30, 40 and 100 year equivalence ratios are 44, 30, 23 and 10 respectively, yet the coefficients of variation are 0.32, 0.33, 0.34, and 0.36 respectively.

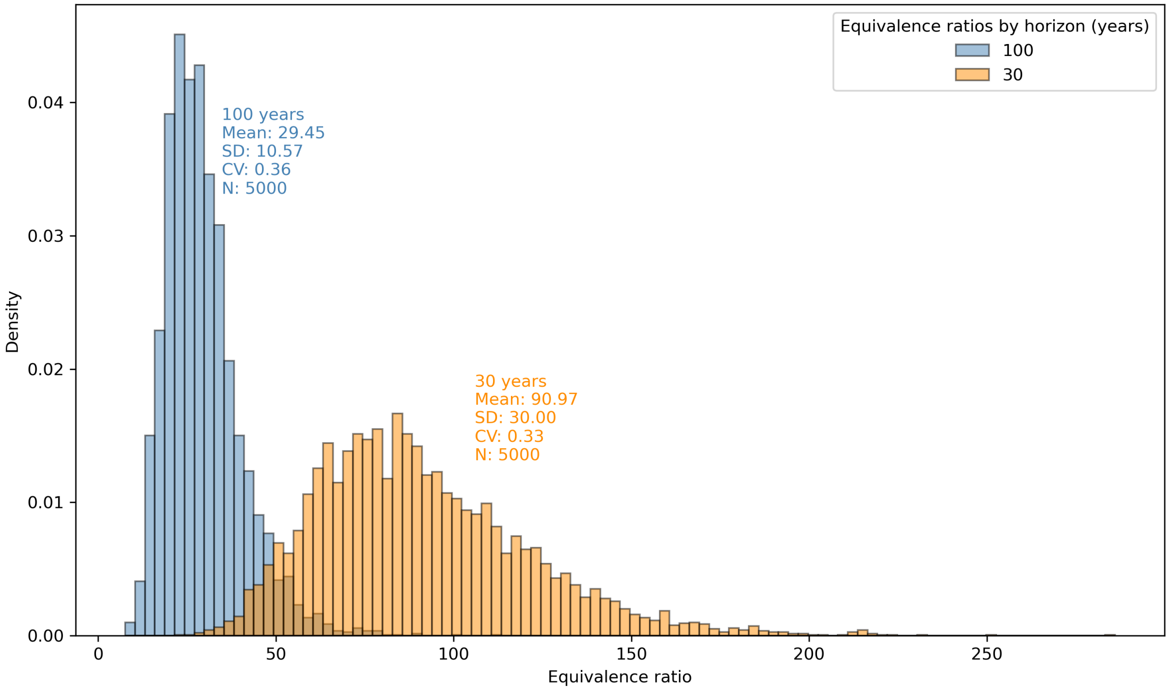

Figure S3. Uncertainty of the equivalence ratio. Histogram of the number of tonnes of CO_2 removed during 30 years (orange) or 100 years (green) which are welfare equivalent to one emitted tonne of CH_4. Discount rate 2.5%, quadratic damages proportional to GDP, growth rate of GDP 2%, no failure risk. There is a lower mean value of equivalence for longer time horizons, yet while the standard deviation is larger for the short-horizon, the coefficient of variation is lower: 0.33 versus 0.36 for the long horizon.

References

1. Leach, N. J. *et al.* Fairv2.0.0: a generalized impulse response model for climate uncertainty and future scenario exploration. *Geosci. Model. Dev.* **14**, 3007–3036, DOI: 10.5194/gmd-14-3007-2021 (2021).
2. Joos, F. *et al.* Carbon dioxide and climate impulse response functions for the computation of greenhouse gas metrics: a multi-model analysis. *Atmospheric Chem. Phys.* **13**, 2793–2825, DOI: 10.5194/acp-13-2793-2013 (2013).
3. Geoffroy, O. *et al.* Transient climate response in a two-layer energy-balance model. part i: Analytical solution and parameter calibration using cmip5 aogcm experiments. *J. climate* **26**, 1841–1857 (2013).
4. Groom, B. & Venmans, F. The social value of offsets. *Nature* **619**, 768–773, DOI: 10.2139/ssrn.3889093 (2023).
5. Howard, P. H. & Sterner, T. Few and not so far between: A meta-analysis of climate damage estimates. *Environ. & resource economics* **68**, 197–225 (2017).
6. Masson-Delmotte, V. *et al.* (eds.) *Climate Change 2021: The Physical Science Basis. Contribution of Working Group I to the Sixth Assessment Report of the Intergovernmental Panel on Climate Change* (Cambridge University Press, Cambridge, United Kingdom and New York, NY, USA, 2021).
